# Improving delayed discharge in gastrointestinal surgery patients: An integrative review

**DOI:** 10.1016/j.ijnsa.2025.100417

**Published:** 2025-09-08

**Authors:** Mathulada Chaimee, Jutharat Attawet, Yunjing Qiu, Thomas J Hugh, Pauline Murray-Parahi, Amanda Wilson

**Affiliations:** a School of Nursing and Midwifery, Faculty of Health, University of Technology Sydney, Sydney, Australia; b Gastrointestinal Surgery and Gastroenterology Unit, Royal Northshore Hospital, Sydney, Australia; c School of Health Sciences, Department of Nursing, Swinburne University of Technology, Hawthorn, Australia; d Surgery Sydney Medical School - Northern, The Sydney University, Sydney, Australia; e Upper Gastrointestinal Surgery Department, Royal North Shore Hospital, Sydney, Australia; f School of Nursing and Midwifery, Faculty of Health, Charles Darwin University, Sydney campus, Sydney, Australia; g Head of School and Dean of Nursing and Midwifery, School of Nursing and Midwifery / College of Health, Medicine and Wellbeing, Newcastle University, Newcastle, Australia; h School of Nursing and Midwifery, Edith Cowin University, Bunbury, South Western Australia, Australia

**Keywords:** Delayed discharge, Surgical patients, Gastrointestinal surgical procedures, Colorectal surgery, Length of stay, Postoperative complications, Postoperative care, Discharge planning, Care transitions, Multidisciplinary care team

## Abstract

**Background:**

Delayed discharge is a global challenge that strains healthcare systems and affects patient outcomes. In gastrointestinal surgery patients, delays often result from a continuum, clinical complications prolong the acute stay and create additional care needs, such as rehabilitation and specialised homecare, which lead to further delays. However, existing literature provides limited insight into this patient group, as most studies generalise the issue. A focused integrative review is therefore needed to synthesise the causes, impacts, and strategies of delayed discharge and to inform more effective discharge planning.

**Objective:**

This review aims to synthesise evidence related to delayed discharge in patients undergoing gastrointestinal surgery. Specifically, it seeks to: (1) identify multi-level contributing factors (patient, clinical, and healthcare system); (2) evaluate significant clinical and economic impacts on patients, family, healthcare staff and healthcare system; and (3) identify and describe effective interventions implemented to promote timely and safe discharge in this population.

**Method:**

A literature search was conducted across CINAHL, Medline, Scopus, Cochrane, and PsycINFO databases for studies published from 2000 to January 2025. Keywords used included “delayed discharge,” “factors,” “impact,” “gastrointestinal surgery,” and “intervention.” Inclusion criteria focused on peer-reviewed studies involving adult gastrointestinal surgery patients in acute hospital settings. Two authors independently screened titles, abstracts, and full texts using the Joanna Briggs Institute SUMARI software. The methodological quality of studies was assessed using the Joanna Briggs Institute critical appraisal tools. Data extraction focused on study characteristics, factors, impacts, and interventions, followed by a deductive narrative analysis to identify patterns and relationships.

**Results:**

Of the 572 articles identified, 20 met inclusion criteria: 17 cohort studies (12 retrospective, five prospective), one analytic cross-sectional study, and two clinical trials. The findings encompassed the length of stay and delayed discharge rate, contributing factors, impacts, and potential interventions. Delayed discharge stemmed from patient, surgical, and system-related factors, affecting patients and hospital efficiency. Targeted interventions, like nurse-led stoma education and streamlined discharge criteria, significantly reduced delays (*p* < 0.0001).

**Conclusion:**

Delayed discharge in gastrointestinal surgery patients stems from a complex interplay of patient, surgical, and systemic factors, affecting both individual and healthcare system. Evidence supports nurse-led and multidisciplinary approaches in mitigating delays, improving outcomes and enhancing healthcare efficiency. Future research should employ qualitative or mixed-methods approaches to explore the psychosocial impacts on patients, families, and healthcare staff, in collaboration with comprehensive, multidisciplinary discharge strategies.

**What is already known?**

• Limited pre-operative education: Inadequate preparation for stoma care, wound management, and dietary changes increases patient anxiety, support needs, and complication risks, delaying the transition to home care.
• Challenges in discharge planning: Logistical barriers, including rehabilitation arrangements, access to home healthcare services, follow-up scheduling, and procurement of medical equipment, frequently impede timely discharge.
• Variability in enhanced recovery after surgery implementation: Enhanced recovery after surgery protocols optimise pre-operative care, promote early mobilisation, and minimise narcotic use to shorten hospital stays and reduce discharge delays. However, deviations from protocol remain common, affecting outcomes.

**What does this paper add?**

• Nurse-led interventions, in collaboration with multidisciplinary teams, significantly improve discharge efficiency.
• Nurse-led discharge interventions, focusing on preoperative education and structured discharge criteria enhance the timeliness of discharge for gastrointestinal surgery patients.
• Tailoring effective post-operative care strategies reduce complications and psychological distress, supporting smoother transitions to home care and minimising delays.

## 1 Introduction

Delayed discharge is a significant global issue, affecting patients, healthcare workers, and healthcare systems (Cadel et al., 2021). During the COVID-19 pandemic, increased bed occupancy further highlighted the need for effective bed management (van den Ende et al., 2023). International data show the magnitude of this problem, with between 10 % and 24 % of hospital beds in high-income countries occupied by patients who no longer require acute care (American Hospital Association, 2022b; Ibrahim et al., 2022; van den Ende et al., 2023). In the United Kingdom, approximately one in five patients in community hospitals experience delays in discharge (NuffieldTrust, 2024). In Australia, approximately 13 % of patients in the Intensive Care Unit experience discharge delays, and similarly, 17 % of surgical patients in New Zealand encounter delayed discharge (Forster et al., 2020; Health and Disability Commissioner, 2022). These figures illustrate the persistent global challenge of delayed discharge and highlight the need for targeted interventions to enhance patient flow and optimise resource allocation.

In clinical care, delayed discharge occurs when a patient remains in hospital despite being medically fit to leave, usually due to non-clinical barriers such as limited community services, rehabilitation beds, or inadequate social support (El-Abbassy et al., 2021; Houghton et al., 2016). This is distinct from prolonged hospitalisation, which results from ongoing medical needs (Tefera et al., 2020). Although both lead to longer hospital stays, delayed discharge reflects primarily non-clinical barriers, while prolonged hospitalisation is driven by clinical necessity.

This distinction is especially relevant in gastrointestinal surgery, where patients face both complex clinical needs and system-level barriers. Post-surgical complications such as ileus, anastomotic leakage, or wound infections may prolong hospitalisation (Emmanuel et al., 2017; Karunakaran et al., 2020). Even after stabilisation, discharge can still be delayed due to challenges such as stoma education, wound management, or lack of rehabilitation access (Parini et al., 2023). Thus, delayed discharge in gastrointestinal surgery often lies at the intersection of clinical complexity and organisational constraints, with significant consequences for patients, caregivers, staff, and health services.

In practice, delayed discharge in gastrointestinal surgery patients often results from a continuum: clinical complications prolong the acute stay and create new care needs that, if not met, lead to delayed discharge (Guadagni et al., 2024; Houghton et al., 2016). Factors contributing to this transition include advanced age, comorbidities, frailty, surgical complications, and gaps in system coordination, such as rehabilitation or discharge planning (Caminsky et al., 2021; Kumar, 2022; Pellico-López et al., 2022). Regardless of cause, extended hospitalisation is associated with adverse outcomes, including physical deconditioning, emotional distress, caregiver strain, staff burnout, and increased healthcare costs (Kritikou et al., 2016; Rawal et al., 2019; Rojas‐García et al., 2018).

Despite recognition of these issues, current literature provides limited insight into delayed discharge specific to gastrointestinal surgery, with much research either generalising the problem or overlooking this patient group. In this review, we defined causes as patient, clinical, and healthcare system factors that contribute to delayed discharge (e.g., advanced age, frailty, post-surgical complications, limited rehabilitation access). We defined consequences as the outcomes of delayed discharge, including clinical harms (e.g., deconditioning and infection), psychosocial effects (such as distress and caregiver strain), and system-level impacts (e.g., insufficient bed flow and increased costs). This integrative review synthesises evidence on the causes, consequences, and interventions addressing delayed discharge in gastrointestinal surgery patients.

## 2 Objective

This integrative review aims to synthesise evidence related to delayed discharge in patients undergoing gastrointestinal surgery. Specifically, it seeks to: (1) Identify contributing factors at the patient, clinical, and healthcare system levels. (2) Evaluate the clinical and economic impacts on patients, families, staff, and the healthcare system. (3) Identify and describe effective interventions to promote timely and safe discharge in gastrointestinal surgery patients.

## 3 Methods

### 3.1 Design

This study employs an integrative review methodology. An integrative literature review effectively synthesises evidence to provide a comprehensive and structured understanding of complex issues (Whittemore and Knafl, 2005). This approach is particularly valuable when research on a topic is limited or when studying a new phenomenon, as it combines diverse empirical (quantitative and qualitative) and theoretical studies to generate insights and guide future research (Cronin and George, 2023). In nursing and health sciences, this methodology is valuable for consolidating existing knowledge to inform practice, policy, and future research by providing a holistic perspective on a specific phenomenon (Hopia et al., 2016).

An integrative review methodology was selected to address the multifaceted nature of delayed discharge in patients undergoing gastrointestinal surgery. This phenomenon involves a complex interplay of patient-level factors, clinical considerations, family dynamics, and broader healthcare system influences. Given that our review aims to synthesise evidence on contributing factors, impacts across multiple stakeholders (including patients, families, healthcare staff, and the healthcare system), and strategies to support timely and safe discharge, an integrative review is well-suited. Unlike scoping reviews, which primarily map the breadth of existing literature, integrative reviews enable a more comprehensive and critical synthesis of diverse methodologies (Torraco, 2005; Whittemore and Knafl, 2005), thereby generating a deeper understanding of delayed discharge in the context of patients undergoing gastrointestinal surgery, informing both clinical practice and future research.

### 3.2 Search strategy

A three-step search strategy was formulated in collaboration with a librarian to identify all relevant published research. The initial step involved an informal exploratory search in MEDLINE (EBSCOhost) and PubMed to find articles relevant to the subject. Important terms, including medical subject headings (MeSH terms) and keywords from the titles and abstracts of these articles, were gathered to create a fundamental logic grid. The search terms were further refined using the PICO framework to ensure they aligned with the study's research questions. The concepts, terms, and phrases from this grid were then used to develop a comprehensive search strategy for MEDLINE (EBSCOhost; see Table 1). The strategy was also adapted for use with various other databases and information sources, including CINAHL (EBSCOhost), Scopus, Cochrane, and PsycINFO.

**Table 1:** PICO tool for question formulation and keywords.

Element	Definition for DHD in GI surgery	Keywords
P (Population)	Patients undergoing GI surgery	“gastrointestinal surgery" OR “GI surgery" OR “abdominal surgery" OR "upper gastrointestinal surgery" OR "colorectal surgery" OR “Gastric surgery” OR “Hepatobiliary surgery” OR “Pancreoduodenal surgery”
I (Intervention)	No direct intervention; instead, identifying barriers to timely discharge and intervention for timely discharge	factors, cause, barriers, influence, “factors contributing," AND intervention OR strategy OR policy OR "best practice."
O (Outcome)	Delayed hospital discharge, its impacts	“delayed discharge” OR "delayed hospital discharge" OR "discharge delay" OR "timely discharge " OR “altered level of care” AND impact OR effect OR outcome OR "healthcare utilisation" OR "patient outcome" OR "staff workload"
C (Comparison	N/A	N/A

The database search was conducted using CINAHL (EBSCOhost), Medline (EBSCOhost), Scopus, Cochrane, and PsycINFO. Keywords were searched across all databases, using synonyms, alternative spellings and truncation to ensure comprehensive retrieval of relevant studies. Boolean operators (“AND,” “OR,” and “NOT”) were used to refine and structure the search strategy effectively. See example search strings in Box 1.

Box 1 **Example search strings**

(“delayed discharge” OR " delayed hospital discharge" OR "discharge delay" OR "timely discharge " OR “altered level of care”) (S1) AND ("gastrointestinal surgery" OR "GI surgery" OR "abdominal surgery" OR "upper gastrointestinal surgery" OR "colorectal surgery" OR “Gastric surgery” OR “Hepatobiliary surgery” OR Pancreoduodenal surgery”) (S2) AND (factors, cause, barriers, influence, “factors contributing" (S3) AND (impact OR effect OR outcome OR "healthcare utilisation" OR "patient outcome") (S4) AND (intervention OR strategy OR policy OR "management practice") (S5)

**S1 AND S2 AND S3**

**S1 AND S2 AND S4**

**S1 AND S2 AND S5**

Alt-text: Unlabelled box

### 3.3 Inclusion and exclusion criteria

This review focused on adult patients undergoing gastrointestinal surgery, both upper and lower gastrointestinal systems, who experienced delayed discharge in a hospital ward setting. The initial search was conducted in April 2024, covering studies published from 2000 until April 2024. The decision to use 2000 as the starting point was based on two factors: delayed discharge has become a significant issue over the past three decades (Micallef et al., 2022); that the widespread adoption of laparoscopic (keyhole) surgery since 2000 has resulted in a significantly lower length of stay in surgical patients (Vignali et al., 2021). To ensure the inclusion of the most recent and relevant studies, a second search was conducted in mid-January 2025. Two additional databases (Cochrane and PsycINFO) were added to enhance the search’s comprehensiveness. The inclusion and exclusion criteria for studies are outlined in Table 2.

**Table 2:** Inclusion and exclusion criteria.

Item	Inclusion criteria	Exclusion criteria
Year of publication	2000 – Mid-January 2025	Prior 2000
Type of Study	Peer-reviewed primary research (quantitative, qualitative, mixed method, clinical trials)	Expert opinion, letters, reports, editorials, grey literature, and literature reviews to avoid duplication and redundancy.
Surgical types	Laparoscopic and laparotomy surgery	Diagnostic procedures such as endoscopy, gastroscopy, colonoscopy, diagnostic laparoscopy
Research setting	Hospital setting; surgical wards	Other hospital settings (ICU, PACU, emergency, outpatient clinics, day surgeries), medical centres, or rehabilitation.
Research Participants	Adults 18 years and over who had gastrointestinal surgery	Paediatric participants (under 18 years) - Other types of surgical procedures
Publication Language	English	Non-English

#### 3.3.1 Justification of inclusion and exclusion criteria

Studies assessing a general surgical population were reviewed for their relevance to patients undergoing gastrointestinal surgery. Only those with clearly identifiable data specific to gastrointestinal surgery were included. Studies were evaluated to determine whether results for patients undergoing gastrointestinal surgery were reported separately or could be extracted from available data. If the gastrointestinal surgery subgroup was not adequately defined or the data could not be separated, the study was excluded from the analysis. This approach ensured that the results were directly relevant to patients undergoing gastrointestinal surgery, and the research in this area focused on delayed discharge in this population.

Grey literature can be used as a supplementary source in large-scale syntheses. However, its inclusion poses challenges due to the lack of systematic methodologies and inconsistencies in assessing quality (Godin et al., 2015). Given these limitations, grey literature was excluded from this review to minimise publication bias and enhance the quality and reliability of findings.

Paediatric patients differ significantly from adults in terms of physiology and recovery, necessitating distinct discharge planning approaches. A study of hospital discharge processes found that children have unique care needs compared to adults, requiring fundamentally different discharge planning (Omonaiye et al., 2024). Including paediatric populations would introduce additional heterogeneity, potentially complicating analyses of discharge difficulties in adult gastrointestinal surgery patients. Therefore, this review focused exclusively on adult patients to maintain a more homogenous study population.

Discharge planning varies significantly across hospitals. While discharge processes in hospital wards are generally straightforward, those in ICUs are more complex due to the higher level of care and need for specialised intervention (Gonçalves-Bradley et al., 2022). By focusing on patients in a surgical ward setting, this review ensures a more homogeneous study population, allowing for a clearer understanding of discharge dynamics relevant to routine postoperative care. Excluding non-adult populations and non-ward settings enhances the specificity of the study, increasing the practical and theoretical relevance of the findings for adult patients undergoing gastrointestinal surgery in hospital wards.

### 3.4 Study selection

Studies were retrieved through database searches and then imported into EndNote™21. (Clarivate, 2023) to remove duplicate entries. The remaining articles were exported to the Joanna Briggs Institute (JBI) SUMARI software (JBI, 2014) for systematic screening. Study selection was conducted by two independent reviewers who assessed the titles, abstracts and full-text articles based on the inclusion and exclusion criteria. Both reviewers independently evaluated all the studies, and any disagreements were resolved through in-person discussion. If consensus could not be reached, a third reviewer was consulted. This approach ensured that the article selection process was systematic and transparent.

### 3.5 Methodological quality and risk of bias assessment

The primary purpose of this integrative review is to synthesise diverse research studies to achieve a comprehensive understanding of delayed discharge in patients undergoing gastrointestinal surgery. To ensure the trustworthiness of this synthesis, it is essential to evaluate both methodological quality and potential risk of bias in each included study. Although related, these processes are distinct. Methodological quality appraisal evaluates how well a study was designed and conducted, thereby strengthening the validity of the conclusions by minimising systematic error (Higgins et al., 2011; Toronto and Remington, 2020). Risk of bias assessment focuses on potential methodological flaws that could distort results, thereby informing the reliability of the synthesis by clarifying the degree of confidence that can be placed in the consistency of the findings across studies (Hopia et al., 2016; Toronto and Remington, 2020). Both assessments are therefore crucial in enhancing the overall credibility of the synthesis, as they provide complementary evidence supporting both validity and reliability of the conclusions (Hopia et al., 2016; Toronto and Remington, 2020).

The Joanna Briggs Institute Critical Appraisal Tools (JBI, 2017) were employed for quality appraisal, selecting the specific checklist appropriate for each study design (cohort, analytical cross-sectional, randomised controlled trial, and quasi-experimental). The criteria used for appraisal were the specific questions within each Joanna Briggs Institute critical appraisal tool, which are detailed in Supplementary 1. These criteria systematically assess key domains related to study validity and risk of bias, including: the clarity of inclusion/exclusion criteria; the methods used for participant selection and group comparison; the validity and reliability of how exposures and outcomes were measured; the identification and management of confounding factors; the completeness and handling of follow-up data; and the appropriateness of the statistical analyses used (Munn et al., 2023).

We chose the Joanna Briggs Institute critical appraisal tools to evaluate methodological quality because they provide a systematic way to evaluate methodological rigour across the various study designs found in our review (Munn et al., 2023), including cohort studies, cross-sectional studies, randomised controlled trials, and quasi-experimental designs. Since most studies (17/20) were cohort studies, the cohort tool was particularly valuable for assessing key sources of bias, such as participant selection, confounding factors, and follow-up (Munn et al., 2020).

Following standard guidelines (Moher et al., 2010), two independent reviewers assessed each study using the Joanna Briggs Institute critical appraisal Tools. Each tool has specific criteria, which were scored as ‘Yes’ (met), ‘No’ (not met), ‘Unclear’, or ‘Not Applicable’ (Barker et al., 2023). Any disagreements between the reviewers were resolved through discussion to reach a consensus. Following Franco et al. (2020), we categorised the overall risk of bias based on the percentage of 'Yes' responses: low (≥70 %), medium (50–69 %), or high (≤49 %). The assessment found 17 studies with a low risk of bias and three studies with a medium risk. Detailed risk of bias assessment results are in Table 3.

**Table 3:** Joanna Briggs Institute critical appraisal and risk of bias results.

Table 3.1 Critical Appraisal of Eligible Cohort Study															
Citation	Q1	Q2	Q3	Q4	Q5	Q6	Q7	Q8	Q9	Q10	Q11	%Yes	Risk		
Caminsky et al., 2021.	N/A	N/A	Y	U	Y	Y	Y	N/A	N/A	Y	Y	54.54	Medium		
Chand et al., 2016.	N/A	N/A	Y	N/A	N/A	Y	Y	Y	N/A	Y	Y	54.54	Medium		
Collard et al., 2020.	Y	Y	Y	Y	Y	Y	Y	Y	U	N/A	Y	81.81	Low		
Emmanuel et al., 2017.	Y	Y	Y	U	U	Y	Y	Y	Y	N/A	Y	72.27	Low		
Hallam et al., 2018.	Y	Y	Y	Y	Y	Y	Y	Y	Y	Y	Y	100	Low		
Karunakaran et al., 2020.	Y	Y	Y	U	U	Y	Y	Y	Y	N/A	Y	72.72	Low		
Keller et al., 2017.	Y	Y	Y	Y	Y	Y	Y	Y	Y	Y	Y	100	Low		
Kocian and Whitley 2022.	Y	Y	Y	Y	Y	Y	Y	Y	Y	Y	Y	100	Low		
Lagerros et al., 2020.	Y	Y	Y	N/A	N/A	Y	Y	Y	N/A	N/A	Y	63.63	Low		
Ngui et al., 2011.	Y	Y	Y	U	U	Y	Y	Y	Y	N/A	Y	72.72	Low		
Parise et al., 2020.	Y	N/A	Y	Y	Y	Y	Y	Y	U	N/A	Y	72.72	Low		
Slieker et al., 2017.	Y	Y	Y	U	U	Y	Y	Y	Y	N/A	Y	72.72	Low		
Smart et al., 2012.	Y	Y	Y	N	N	Y	Y	Y	Y	N/A	Y	72.72	Low		
Vignali et al., 2021.	Y	Y	Y	Y	Y	Y	Y	Y	U	N/A	Y	81.81	Low		
Keller et al., 2014.	Y	Y	Y	Y	Y	Y	Y	Y	Y	Y	Y	100	Low		
Boulind et al., 2012.	Y	N/A	Y	Y	Y	Y	Y	Y	U	N/A	Y	72.72	Low		
Claydon et al., 2022	Y	Y	Y	U	U	Y	Y	Y	Y	Y	Y	81.81	Low		

Table 3.2 Critical Appraisal of Eligible Analytical Cross-Sectional Study															
Citation	Q1	Q2	Q3	Q4	Q5	Q6	Q7	Q8	%Yes	Risk					
Munk-Madsen et al., 2019.	Y	Y	Y	Y	N	N	Y	Y	75	Low					

Table 3.3 Critical Appraisal of Eligible Quasi-Experimental Study															
Citation	Q1	Q2	Q3	Q4	Q5	Q6	Q7	Q8	Q9	% Yes	Risk				
Younis et al., 2012.	Y	Y	Y	Y	N	Y	Y	Y	Y	88.88	Low				

Table 3.4 Critical Appraisal of Eligible Randomized Controlled Trial															
Citation	Q1	Q2	Q3	Q4	Q5	Q6	Q7	Q8	Q9	Q10	Q11	Q12	Q13	%Yes	Risk
Forsmo et al., 2016.	Y	N	Y	N	N	N	Y	Y	U	Y	Y	Y	Y	61.5	Medium

By integrating these appraisals into the synthesis, we ensured that studies with stronger methodological quality contributed more directly to the validity of our conclusions, while careful consideration of studies with some risk of bias allowed us to evaluate the reliability and consistency of the overall evidence base. No studies were excluded based on quality scores. Consistent with the integrative review methodology and the Joanna Briggs Institute guidelines, the quality appraisal and bias assessment were used to inform the synthesis and interpretation of the findings (Barker et al., 2023). The assessments served as a lens to evaluate the trustworthiness of the included evidence. Findings from studies assessed as low risk of bias were given greater interpretive weight, whereas results from medium-risk studies were approached with appropriate caution (Barker et al., 2023)

### 3.6 Data extraction

Twenty articles were extracted by two independent reviewers using a standardised Joanna Briggs Institute extraction form. This form captured standard publication details (year, location, design), participant characteristics (age, gender, diagnosis), and, crucially for the synthesis, the specific reported findings related to delayed discharge, study limitations, and any author recommendations (see details in Supplementary 2 and 3). To ensure consistency and accuracy, inter-rater reliability checks were conducted by comparing the extracted data between the two reviewers. Any discrepancies were resolved through discussion, with consultation of the original studies where necessary. All authors reviewed the extracted data for added verification. Regarding missing data management, the initial plan was to contact the first authors via email to request the missing outcome data and discuss the potential impact of the missing data on the review findings. However, during the data extraction phase from the 20 included studies, we found that all essential outcome data to achieve the review’s objective were adequately reported.

### 3.7 Data synthesis

A deductive narrative analysis approach was employed to guide the data synthesis, ensuring a systematic, transparent, and conceptually coherent interpretation of the literature (Younas et al., 2022). This method allowed the authors to structure the analysis around a set of predefined categories aligned with the review objectives and informed by prior research. Four overarching deductive categories were established before analysis commenced: (1) Length of stay and delayed discharge rates, (2) Factors contributing to delayed discharge, (3) Impacts of delayed discharge, and (4) Interventions aimed at promoting timely and safe discharge.

Guided by a pre-defined analytical framework consisting of four main categories. The primary objective was to sort and map the evidence from the selected studies onto these existing categories. To achieve this, two reviewers began by extracting relevant findings from the results and discussion sections of each paper. These findings included reported statistics on length of stay and delayed discharge, specific risk factors (e.g., age, comorbidity, surgical complications), consequences (e.g., increased patient anxiety, additional bed-day costs), and details of interventions.

Each extracted piece of data was then systematically coded and allocated to one of the four pre-defined categories. Following this, the reviewers analysed the data collated within each category to synthesise the narrative subcategories (Hecker and Kalpokas, 2024). For instance, under the main category *‘Factors contributing to delayed discharge,’* findings were further organised into pre-determined sub-categories such as *‘patient factors,’ ‘clinical factors,’ and ‘system issues.’* This structured coding allowed specific details from the literature, such as ‘ileus,’ ‘anastomotic leak,’ and ‘wound infection’, to be logically grouped under the *‘post-operative clinical complications’* sub-category. This process did not involve generating new themes but rather populating the deductive structure with specific evidence from the literature. Next, all authors collaboratively reviewed the entire synthesis to ensure the data were consistently and logically organised within the established framework, with primary articles revisited as needed for confirmation. A summary of key outcomes is presented in Table 4.

**Table 4:** Summary outcomes in gastrointestinal surgery studies.

Category	LOS and delayed discharge Rate	Factors contributing to delayed discharge	Impacts of delayed discharge	Interventions to improve timely discharge
**LOS**	Median LOS under ERAS protocol: 3 to 6 days	Pre-existing co-morbidities (e.g., psychiatric conditions, chronic pain)	Increased rates of postoperative complications	Nurse-focused stoma counselling and education program
				
	Median LOS deviating from ERAS protocol: 7+ days	Pre-operative factors (age, BMI, living in a retirement village)	Higher readmission rates	Simple discharge criteria
				
		Chemotherapy or radiotherapy	Increased LOS	Pre-operative stoma education within the ERAS program
				
**Delayed discharge Rate**	DHD rate ranged from 15 % to 72 %	Intra-operative complications (e.g., conversion from laparoscopic to laparotomy surgery)	Increased healthcare costs (per bed/day)	
				
		Operation complexity and duration	Impact on hospital bed flow efficiency	
				
		Post-operative complications (e.g., ileus, infections)		
				
		Delayed post-operative recovery (e.g., need for stoma education, lack of early mobilisation)		
				
**Healthcare Factors**		Patient refusal to discharge		
				
		Systematic issues (e.g., team reluctance due to no clear discharge criteria, ongoing treatment needed)		
				
		Resource limitations (e.g., rehabilitation, home care)		

* LOS = Length of stay

* ERAS = Early recovery after surgery

## 4 Results

### 4.1 Search results

**Fig. 1:**
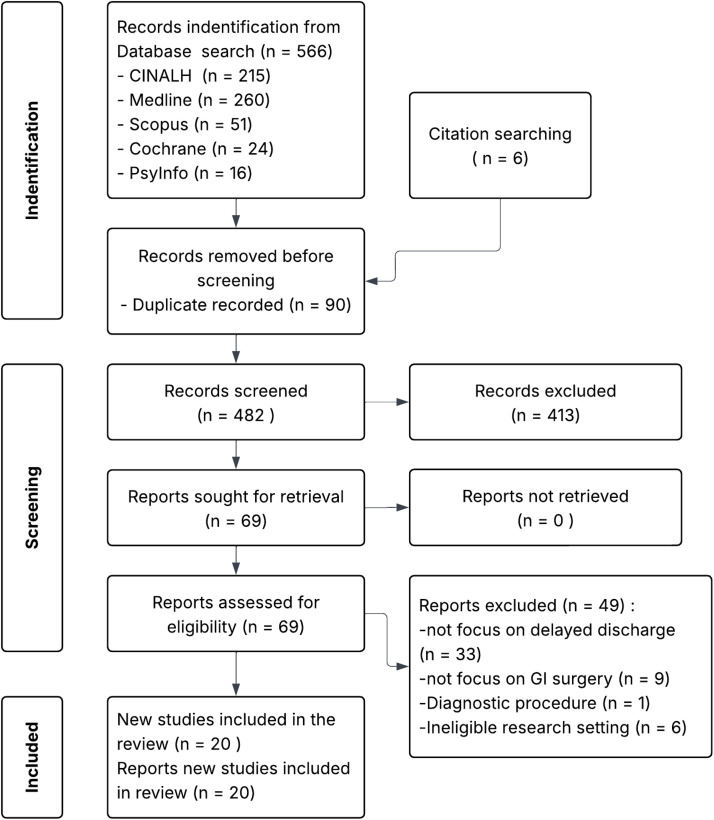
Prisma flowchart.

The initial search identified 572 articles, including six relevant articles identified through references and citation tracking. After removing duplicate articles and screening titles, abstracts, and full texts, 20 articles were included for critical appraisal. All 20 articles were included in the review, as 17 had a low risk of bias, and three had a medium risk, indicating moderate to high quality. Of these, 17 were cohort studies (12 retrospective and five prospective), one was an analysis–cross–sectional study, and two were clinical trial studies (one randomised controlled trial and one quasi-experimental study). See Fig. 1: PRISMA Flowchart for search details.

### 4.2 Characteristics of included studies

This review included 20 quantitative studies involving 27,746 participants. The mean participant age ranges from 60 to 75. Fifteen studies were conducted in European countries, two in the United States and one each from Canada, Australia, and India. The majority of the included studies (*n* = 16) focused on colorectal surgery, with four addressing upper gastrointestinal surgery. The procedures included colorectal resections, pancreatoduodenectomy, gastrectomy, gastric bypass, and appendicectomy. See Supplementary 2 and 3 for details of included studies.

### 4.3 Outcomes

The findings were categorised into four key areas: (1) Length of stay and delayed discharge rates, (2) factors contributing to delayed discharge, (3) impacts of delayed discharge, and (4) interventions to improve timely discharge.

#### 4.3.1 Length of stay and delayed discharge rates

Fifteen of 20 studies reported the length of stay. The median length of stay ranged from three to six days under the enhanced recovery after surgery protocol, while deviations from the protocol resulted in length of stay extending to seven days or more. The median length of stay for non-delayed discharge patients was six days, while delayed discharge patients had a median length of stay exceeding seven days. Sixteen studies reported delayed discharge rates, which ranged from 15 % to 72 %. (Boulind et al., 2012; Collard et al., 2020; Keller et al., 2014). See Supplementary 3 for details on length of stay and delayed discharge rates.

#### 4.3.2 Factors contributing to delayed discharge

Nine studies examined **patient-specific factors** contributing to delayed discharge. These included pre-existing comorbidities such as psychiatric conditions (Lagerros et al., 2020), anxiety, and chronic pain (Keller et al., 2017) as well as factors like age, body mass index (BMI) (Chand et al., 2016; Keller et al., 2014), and residing in a retirement village (Keller et al., 2014; Ngui et al., 2011). Patients aged 70 years and older had a longer length of stay and higher delayed discharge rates than younger patients (Boulind et al., 2012; Ngui et al., 2011). Similarly, those who had recently received chemotherapy or radiotherapy had the longest length of stay and the highest delayed discharge rates (Hallam et al., 2018). However, Hallam et al. (2018) found that age alone was not a significant factor in delayed hospital discharge. Psychosocial factors also played a role, with 16 % requesting further observation and 11 % expressing a reluctance to be discharged, contributing to delays (Caminsky et al., 2021; Collard et al., 2020).

##### 4.3.2.1 Surgical Factors

Ten studies identified surgical factors related to delayed discharge in gastrointestinal surgery patients. These included the type of operation, intraoperative complications, operation duration, and postoperative complications. Patients undergoing laparotomy and complex operations, such as conversion to stoma formation, had higher delayed discharge rates compared to those who had laparoscopic surgery (Emmanuel et al., 2017; Hallam et al., 2018; Kocián and Whitley, 2022). Intraoperative complications, including blood loss, blood transfusion, and failure to extubate postoperatively, were associated with higher delayed discharge rates (Collard et al., 2020; Karunakaran et al., 2020; Keller et al., 2014; Keller et al., 2017; Parise et al., 2020). Longer operation time also led to delayed discharge (Claydon et al., 2022; Parise et al., 2020). Additionally, postoperative complications such as paralytic ileus, anastomotic leakage, abdominal collection, abdominal pain, and wound infections were significant contributors to delayed discharge (Emmanuel et al., 2017; Karunakaran et al., 2020; Kocián and Whitley, 2022; Smart et al., 2012).

##### 4.3.2.2 Healthcare system factors

Nine studies found that healthcare system-related factors, including resource limitations, care delivery processes, and policy issues, contribute to delayed discharge in patients undergoing gastrointestinal surgery. Slieker et al. (2017) noted that a lack of rehabilitation services, aged care facility placements, stoma education, or family members available to care for the patient at home contributed to 20 % of delayed discharge cases. Delays in care delivery included failure to mobilise patients early postoperatively (Boulind et al., 2012; Kocián and Whitley, 2022; Parise et al., 2020) and late cessation of intravenous fluids, delayed feeding, and postponed removal of indwelling catheters (IDC) (Boulind et al., 2012; Smart et al., 2012; Vignali et al., 2021) associated with delayed discharge. Reluctance to discharge patients due to a lack of clear discharge criteria (Caminsky et al., 2021) and patient refused to leave the hospital (Collard et al., 2020) further complicate discharge processes, highlighting decision-making and policy challenges in healthcare.

#### 4.3.3 Impacts of delayed discharge

Five included studies reported the impacts resulting from delayed discharge, which we grouped into two domains: individual impacts *and* system impacts. Individual impacts refer to consequences for patients, families, and staff, including clinical harms (e.g., deconditioning, infection), psychosocial distress, caregiver burden, and staff burnout. System impacts capture broader organisational and economic consequences, such as inefficient bed use, higher costs, workforce pressures, and disrupted patient flow. This distinction ensured outcomes were synthesised consistently at both micro and macro levels.

A key challenge in interpreting these findings is separating the consequences of delayed discharge itself (e.g., medically fit patients remaining in hospital) from those of the underlying clinical conditions or complications that initially prolonged hospitalisation and may also contribute to delay. Across the included studies, impacts were observed at both the individual and system levels, underscoring the multifaceted nature of delayed discharge.

Three out of five studies reported impacts of delayed discharge on an individual’s adverse clinical outcomes. For instance, Emmanuel et al. (2017) observed that patients undergoing gastrointestinal surgery who experienced delayed discharge also had higher readmission rates (17 % vs. 12 %) and reoperation rates (18 % vs. 5 %) compared to those discharged on time. Similarly, increased mortality was noted in conjunction with delayed discharge in patients undergoing colorectal cancer resections (Collard et al., 2020; Karunakaran et al., 2020). While these correlations are important, it is plausible that the initial clinical complexities that led to a prolonged stay and subsequent delayed discharge were also significant contributors to these poorer clinical outcomes, rather than delayed discharge being the sole or primary cause.

While other direct individual impacts of delayed discharge, such as patient deconditioning or psychological distress, were not prominently identified in these included studies, the evidence on increased costs and reduced efficiency underscores the significant burden imposed by delayed discharge.

Two out of five studies reported impacts on the healthcare system’s economy and efficiency, which appear to be a more direct impact of delayed discharge itself. Keller et al. (2017) reported that patients experiencing delayed discharge incurred higher costs than those with non-delayed discharge (USD 22,127 vs USD 13,030). Furthermore, delayed discharge was found to negatively affect hospital bed flow efficiency, contributing to increased overall healthcare costs and resource strain (Boulind et al., 2012). These findings suggest that occupying an acute bed beyond medical necessity has tangible financial and operational consequences for healthcare systems.

#### 4.3.4 Interventions to improve timely discharge

Three studies examined interventions designed to improve the timely and safe discharge of patients undergoing gastrointestinal surgery. Nurse-led stoma counselling and education programs were found to significantly reduce length of stay (*p* < 0.0001) (Forsmo et al., 2016). Similarly, preoperative stoma education and the implementation of an enhanced recovery program significantly reduced discharge delays (*p* < 0.0001) (Younis et al., 2012). Emmanuel et al. (2017) found that using simple discharge criteria within 72 hours of colorectal cancer resection surgery significantly reduced postoperative complications and readmission rates (*p* < 0.0001). Refer to Table 5 for details on the interventions and their outcomes.

**Table 5:** Studies focused on interventions to improve DHD rates.

Authors (year)	Participants	Intervention	Results
Forsmo et al. (2016)	122 patients who received a planned stoma	Extensive stoma education course	LOS was significantly shorter in the education group (median 6 days vs. 9 days; *p* < 0.001). Similar outcomes regarding major/minor morbidity, readmission rate, stoma-related complications, and 30-day mortality.
Younis et al. (2012)	240 elective anterior resection with a loop ileostomy pts: 120 prior ERP and 120 post-ERP	Stoma education with ERP	Before ERP: average LOS 14 days (7–25 days); after ERP: average LOS 8 days (3–17 days) (p = 0.70). Delayed discharge due to delay in stoma management: 17.5 % (pre-ERP) vs. 0.8 % (post-ERP) *p* < 0.0001.
Emmanuel et al., 2017	256 colorectal cancer resections (90 % laparoscopic); mean age 71 years	Accelerated discharge with simple discharge criteria	Patients discharged within 72 h had significantly fewer postoperative complications (8 % vs 42 %), readmissions (12 % vs 17 %), and reoperations (3 % vs 18 %) *p* < 0.0001.

* ERP = Enhanced recovery program

## 5 Discussion

This review highlights the multifaceted nature of delayed discharge in patients undergoing gastrointestinal surgery, driven by patient-specific, surgical, and healthcare system factors. The review’s results directly address the research aims and objectives by synthesising the factors contributing to delayed discharge, evaluating its clinical and economic impacts, and identifying effective interventions to promote timely discharge. Importantly, the interpretation of findings was informed by methodological quality and risk of bias assessments. Studies assessed as low risk of bias were given greater interpretive weight, while findings from medium-risk studies were considered with caution. This approach strengthened the validity and reliability of the conclusions and ensured that recommendations were based on the most trustworthy evidence.

### 5.1 Factors contributing to delayed discharge

The findings of this review indicate that patient-specific, surgical, and healthcare system factors contribute to delayed discharge in patients undergoing gastrointestinal surgery.

Patient-specific factors play a crucial role in determining the risk of delayed discharge. Although advanced age is often linked to delayed discharge (Karunakaran et al., 2020; Kocián and Whitley, 2022; Lagerros et al., 2020; Menendez et al., 2019), some studies suggest that age alone is not a direct factor in delayed discharge (Hallam et al., 2018); Houghton et al. (2016). This review's findings support existing evidence that older adults are at greater risk of delayed discharge, particularly when they lack stable housing or require ongoing rehabilitation and home care support. However, our analysis indicates that frailty is a more precise and meaningful predictor than chronological age (Chand et al., 2016; Keller et al., 2017; Lagerros et al., 2020). Frail patients, regardless of age, often have limited physical function, higher levels of dependency, and complex care needs, which further increases the likelihood of delayed discharge, as they need more home care or rehabilitation support (Luis et al., 2023). Therefore, rather than relying solely on age, a comprehensive assessment of a patient's frailty, physical state, and complex care needs provides a more accurate framework for understanding and preventing delayed discharge.

Psychosocial factors, including anxiety and reluctance to leave the hospital, also influence delayed discharge in surgery patients. Some patients fear inadequate pain management or potential postoperative complications, causing them to prefer to stay in hospital (Caminsky et al., 2021). Preoperative anxiety can slow postoperative recovery and increase length of stay (Mancuso et al., 2018). Frail patients or those living alone may be reluctant to leave the hospital due to concerns about inadequate support at home (Lagerros et al., 2020). The absence of family caregivers or suitable post-discharge arrangements further contributes to extended delays (Slieker et al., 2017). Studies also indicate that a large proportion of patients (37 %) refuse discharge despite being medically fit, opting for continued conservative management in hospital (Zhao et al., 2022). These findings highlight the need for patient-centred discharge planning that accounts for psychological and social requirements to enhance the smooth transition from hospital to home or post-acute care services.

Several strategies can address patient-specific barriers. Preoperative education can set clear expectations regarding timely discharge and reduce anxiety. Educating patients about the discharge process improves confidence in recovery and facilitates a smoother transition home. Mindfulness-based cognitive behavioural therapy (MBCBT) has been shown to reduce postoperative pain and support recovery (Pester et al., 2023). Structured discharge planning, including early engagement with coordination teams, provides patients with necessary resources and reassurance (Sreeprasarn and Neelapaichit, 2020). A multidisciplinary approach involving nurse navigators and social workers can further enhance patient confidence in managing recovery at home, thereby reducing delayed discharge (Coffey et al., 2019). Shared decision-making among patients, their families, and healthcare providers can enhance adherence to discharge plans and improve patient satisfaction (Omonaiye et al., 2024). Post-discharge home services, including nursing visits and telehealth, enhance patient confidence by addressing common concerns about post-discharge self-care, complications, and pain management (Handiyani et al., 2024). Thus, arranging community nurses and clearly communicating these supports before discharge provides a crucial safety net, easing anxiety, reducing readmission, and improving the patient experience (Al Qadire, 2023). Psychologically prepared patients are more likely to adapt to their new routine quickly, reducing delays caused by reluctance to leave the hospital.

Surgical factors, including procedural complexity, intraoperative complications and postoperative complications (such as anastomotic leaks, paralytic ileus, wound infection and dehiscence), were major causes of delayed discharge in gastrointestinal surgery. Laparoscopic (key-hole) surgery is associated with shorter hospital stays and lower complication rates compared to laparotomy (Andrews et al., 2021). In contrast, emergency surgery often leads to extended length of stay and higher delayed discharge rates due to the complexity of the patients’ conditions and recovery trajectories (Jerath et al., 2020). Intraoperative factors, such as procedure duration and complications, further extend hospital stays. Operations exceeding 240 min are associated with increased postoperative complications and slower recovery (Tassoudis et al., 2011). Typical postoperative adverse effects, including nausea, vomiting, and infection, further delay discharge following both emergency and elective surgeries (Jerath et al., 2020; van Kooten et al., 2021).

Patients with complex care needs, particularly those with postoperative complications, are more likely to experience delayed discharge (Smith et al., 2016). Complications such as paralytic ileus, wound infection, fistula, and abscesses require ongoing medical treatments and close observation, delaying discharge readiness and need more home care services (Kovoor et al., 2021; Mazzotta et al., 2020). For example, paralytic ileus slows gastrointestinal recovery, while anastomotic leakage necessitates reoperation or drainage, further extending hospital stays. Postoperative care needs, such as intravenous fluids, total parenteral nutrition (TPN), stoma management or wound dressings, contribute to delays in discharge if not completed promptly (Emmanuel et al., 2017; Hallam et al., 2018; Karunakaran et al., 2020).

Healthcare system factors also play a significant role in delayed discharge. Previous studies indicated that delays in medical decision-making, late discharge orders, and limited access to essential resources, such as rehabilitation or home care services, contribute to prolonged hospital stays (Silva et al., 2014; van den Ende et al., 2023). Other system-related factors, such as delayed consultant review or incomplete discharge paperwork, also contributed to the delays (Kumar, 2022; Micallef et al., 2022). Hospital policies and staffing levels are key contextual factors that contribute to delayed discharge in patients undergoing gastrointestinal surgery. Rigid discharge protocols, weekend discharges, and extensive documentation requirements frequently prolong hospital stays (Aggarwal et al., 2024). Hospitals with well-defined discharge protocols and strong multidisciplinary team coordination tend to achieve shorter length of stay (Gonçalves-Bradley et al., 2022; Ibrahim et al., 2022; Meo et al., 2018). Poor discharge decision-making processes, for instance, those lacking standardised criteria, have been linked to increased delayed discharge rates (Caminsky et al., 2021; Emmanuel et al., 2017). Additionally, inaccurate initial discharge plans are highly correlated with delayed discharge. Thus, there is a need to ensure that discharge plans are well developed to enable patient transition without delay (Burns et al., 2024).

Staffing levels also influence hospital discharge efficiency. Physician and nursing shortages, as well as the limited availability of allied health professionals such as physiotherapists, pharmacists, social workers and occupational therapists, contribute to delays in postoperative recovery and discharge readiness. Studies show that poor nurse-to-patient ratios lead to prolonged length of stay due to delayed assessments and interventions (Griffiths et al., 2024). Fogg et al. (2021) reported that increasing registered nurse care by 30 min per patient reduced hospital readmission rates by 6 % among individuals with cognitive impairments. Discharge planners and case managers play a crucial role in coordinating care and addressing discharge barriers, particularly for patients requiring post-acute support (Optimum Personnal Care, 2022). Furthermore, research suggests that staff well-being influences hospital discharge efficiency, with better staff conditions associated with lower delayed discharge rates (Ali and Salehnejad, 2020).

Resolving these challenges demands targeted policy reforms and strategic investment in staffing to optimise discharge practices and enhance patient outcomes. Strengthening interprofessional collaboration and streamlining administrative discharge processes can improve hospital efficiency and minimise unnecessary hospital stays. Caminsky et al. (2021) proposed two solutions: (1) early involvement of the discharge planning team to identify and resolve potential barriers, such as timely preparation of discharge paperwork, pre-discharge consultations with a multidisciplinary team and post-discharge placement arrangements, and (2) improving discharge decision-making among clinicians through standardised hospital and surgery-specific discharge criteria.

### 5.2 Impacts of delayed discharge

This review found a strong relationship between delayed discharge and higher rates of readmission, reoperation, and potentially mortality in gastrointestinal surgery patients (Collard et al., 2020; Emmanuel et al., 2017; Karunakaran et al., 2020). Furthermore, it imposed burdens on the economic resources and capacity of the healthcare system (Boulind et al., 2012; Keller et al., 2017).

Delayed discharge prolongs hospitalisation for medically stable patients, exposing them to additional risks. Physically, patients who experience delayed discharge often face prolonged immobility, which can lead to functional decline, muscle deconditioning, and decreased cardiovascular fitness (Walker et al., 2018). This iatrogenic frailty makes the patient more susceptible to falls and pressure injury, less able to perform activities of daily living upon returning home, and more vulnerable to health deterioration (Walker et al., 2018), thereby increasing the likelihood of readmission. Moreover, prolonged hospital stays significantly heighten their risk of hospital-acquired infections (HAIs), such as Methicillin-resistant Staphylococcus aureus (MRSA), Vancomycin-resistant Enterococci (VRE), and Clostridium difficile (C-Diff), due to extended exposure to pathogens and weakened host defences (Bai et al., 2019; Rawal et al., 2019). A patient who has successfully recovered from their initial condition may then develop a new, hospital-acquired complication that requires further treatment, potential reoperation, or leads to a rapid decline culminating in readmission or even mortality.

Beyond physical risks, delayed discharge has profound psychological and social implications. Patients often experience heightened anxiety, stress, and depression due to uncertainty, loss of control, and prolonged separation from their normal lives (Rojas‐García et al., 2018). Additionally, extended hospitalisation fosters institutionalisation, eroding patients’ sense of independence and making reintegration into the community more challenging (Everall et al., 2019). Concurrently, a delayed and uncertain discharge process places significant strain on family and social support systems. Caregiver fatigue can erode the support structure the patient relies on, leading to an unsafe discharge environment and a higher probability of requiring readmission (Everall et al., 2019). Remarkably, these specific psychological and social impacts were not prominently reported in the included studies, suggesting a potential research gap in assessing the experience during delayed discharge within gastrointestinal surgery patients.

Delayed discharge imposes substantial financial strain on healthcare systems by escalating hospital costs and disrupting operational efficiency (Bai et al., 2019). Prolonged hospitalisation contributes to higher expenditure due to extended resource utilisation, additional medical interventions, and increased staffing requirements (American Hospital Association, 2022a). Rojas‐García et al. (2018) found that patients experiencing delayed discharge incurred substantially higher hospital expenses than those discharged on time, mainly due to additional nursing care, extended accommodation, and increased risk of hospital-acquired infections. Beyond direct costs, delayed discharge disrupts hospital bed flow, leading to access blockages in emergency departments (Morley et al., 2018). When beds remain occupied by patients who no longer require acute care, new admissions, including emergency cases and elective surgeries, face delays, exacerbating overcrowding and limiting timely medical interventions (Bai et al., 2019). This inefficiency disrupts patient flow and delays critical medical interventions for incoming patients.

The economic impact extends beyond hospitals to community healthcare services. Patients with delayed discharge may need further community or rehabilitative care, and the lack of such services exacerbates the situation (Slieker et al., 2017). Poor discharge planning increases readmission rates, placing further strain on hospital resources. Emmanuel et al. (2017) reported that delayed discharge was associated with a 15–20 % increase in 30-day readmission rates, further escalating healthcare costs.

Addressing these challenges requires structured discharge planning, transitional care programs, and multidisciplinary collaboration. Bilicki and Reeves (2024), Coffey et al. (2019), and Sullivan et al. (2018) highlight strategies such as early discharge planning, patient education, and post-discharge follow-up appointments to reduce length of stay and lower 30-day readmission rates. In gastrointestinal surgery, preventing delayed discharge demands strong multidisciplinary coordination to develop tailored discharge plans, targeted interventions and effective policies (Ibrahim et al., 2022). Schmidt et al. (2015) underscores the role of patient education in mitigating anxiety, enhancing recovery, and promoting independence, thereby reinforcing the importance of integrating these approaches into discharge planning. Successful implementation of these strategies not only enhances patient outcomes but also alleviates healthcare burdens, reducing hospital congestion and optimising resource allocation (Kifle et al., 2024).

### 5.3 Interventions to facilitate timely discharge

Although delayed discharge presents significant challenges, various interventions have demonstrated effectiveness in facilitating timely discharge. Key interventions, including standardised discharge criteria, nurse-led stoma counselling, and preoperative education, have proven effective in reducing delayed discharge among gastrointestinal surgery patients (Emmanuel et al., 2017; Forsmo et al., 2016; Younis et al., 2012). Clear discharge criteria support clinicians in making timely discharge decisions. At the same time, preoperative education prepares patients physically, mentally and emotionally for postoperative care, reducing complications and ensuring they are fit for discharge. Graham et al. (2012) found that nurse-led discharge groups achieved higher rates of timely discharge than doctor-led groups, largely due to more efficient implementation of discharge criteria. Similarly, Sreeprasarn and Neelapaichit (2020) reported lower delayed discharge rates with proactive, early discharge planning led by home healthcare nurses compared to reactive approaches that only addressed delayed discharge once it occurred.

The success of these interventions depends on factors such as institutional resources, patient engagement, and variations in the healthcare system. Nurse-led stoma education improves patient confidence and reduces complications that may extend hospital stays, though its success relies on adequate staffing and patient adherence (Forsmo et al., 2016). Similarly, preoperative education facilitates the discharge but requires early patient engagement and strong interdisciplinary coordination (Younis et al., 2012). Challenges such as frailty, health status, understanding capacity and lack of adherence to enhanced recovery after surgery guidelines can hinder success (Cochran et al., 2025). Standardised discharge criteria enhance discharge efficiency and reduce readmissions (Emmanuel et al., 2017). However, clinicians' reluctance to discharge patients early and failure to comply with the criteria remain barriers (Caminsky et al., 2021; Emmanuel et al., 2017). Inadequate post-discharge support services can also lead to suboptimal outcomes, highlighting the importance of an interprofessional approach.

Multidisciplinary teams play a crucial role in addressing discharge barriers. Ibrahim et al. (2022) observed that forming a multidisciplinary discharge coordination team that regularly met to resolve discharge issues reduced delayed discharge rates by 41.5 % without increasing readmissions. This emphasises the need for collaboration among healthcare providers, social workers, occupational therapists and administrative staff to streamline discharge processes. Financial incentives and policies, such as the UK’s Community Care (Delayed Discharges) Act, have been demonstrated to enhance partnerships between hospitals and community services. This policy was not strictly implemented, but it helped improve the relationships between acute hospitals and community care providers (Cadel et al., 2021).

This review found three studies reported on interventions, all of which reported positive results. While understanding which interventions are ineffective is important, no studies within our scope reported null or negative results. This gap may reflect publication bias and is a limitation of the current literature. To support a more balanced understanding, future research should report both successful and unsuccessful intervention outcomes.

## 6 Heterogeneity among the included studies

This review exhibits heterogeneity due to variations in study design, sample size, surgical techniques, and geographic settings, which may limit the generalisability of findings. A significant proportion of the included studies utilised retrospective cohort designs, which are susceptible to data collection bias and confounding (Stroup et al., 2000). Furthermore, variations in sample sizes influenced statistical power, potentially affecting the robustness of findings (Lo et al., 2014). Differences in surgical techniques, such as laparoscopic versus laparotomy procedures (Andrews et al., 2021) alongside inconsistencies in enhanced recovery after surgery protocol implementation further contribute to heterogeneity (Gustafsson et al., 2019; Tian et al., 2024). Additionally, the inclusion of studies from different countries introduces heterogeneity due to varying healthcare systems and discharge practices (Abdelhalim et al., 2024). Given these variations, findings should be interpreted with caution, ensuring interventions are adapted to the specific healthcare context in which they are applied.

## 7 Practical Implications for clinical practice and future research

This integrative review consolidates key factors, impacts, and strategies related to delayed discharge in gastrointestinal surgery, providing a foundation for research, clinical practice, and policy development to enhance patient outcomes and healthcare efficiency.

A multidisciplinary approach is vital, with nurse-led interventions central to discharge planning, patient-centred discharge criteria and collaboration among all disciplines. Effective interventions must be tailored to the specific surgical types of gastrointestinal surgery—whether upper gastrointestinal or colorectal procedures—and strengthened through optimised postoperative protocols, enhanced interdepartmental collaboration, and potential financial incentives to support timely discharge.

Further research is essential to deepen understand of the factors driving delayed discharge and its wide-ranging effects on patients, families, healthcare staff, and systemic efficiency, particularly its psychosocial implications. Future studies should adopt qualitative or mixed-methods approaches to capture diverse perspectives and assess the development of targeted, surgery-specific interventions that enhance discharge efficiency and patient care.

## 8 Limitations

This review has several limitations. Although the integrative design enabled inclusion of diverse study types, only quantitative studies were identified, limiting insights into lived experiences and contextual factors that qualitative research might reveal. Most studies focused on lower gastrointestinal surgery, reducing generalisability to upper gastrointestinal procedures. Reporting bias, including publication and selective outcome reporting, could not be formally assessed due to heterogeneity. Finally, synthesising diverse quantitative data relies on transparent discussion rather than statistical testing, which may involve subjective judgement and risk overrepresentation of positive findings.

## Declaration of generative AI and AI-assisted technologies in the writing process

During the preparation of this work, the authors used Grammarly software in order to improve language and readability, with caution. After using this tool, the authors reviewed and edited the content as needed and take full responsibility for the content of the publication.

## Disclosure statement

The authors reported no potential conflict of interest.

## CRediT authorship contribution statement

**Mathulada Chaimee:** Writing – review & editing, Writing – original draft, Validation, Resources, Project administration, Methodology, Investigation, Formal analysis, Data curation, Conceptualization. **Jutharat Attawet:** Writing – review & editing, Supervision, Methodology, Formal analysis, Data curation, Conceptualization. **Yunjing Qiu:** Writing – review & editing, Validation, Supervision. **Thomas J Hugh:** Writing – review & editing, Supervision, Resources, Conceptualization. **Pauline Murray-Parahi:** Writing – review & editing, Formal analysis, Supervision. **Amanda Wilson:** Writing – review & editing, Validation, Supervision, Resources.

## Declaration of competing interest

The authors declare no competing financial, professional, or personal interests that could have influenced the work reported in this manuscript.

## Appendix Supplementary materials

Image, application 1

Image, application 2

Image, application 3
