# Supplementary material for: Improving delayed discharge in gastrointestinal surgery patients: An integrative review
Source: Int J Nurs Stud Adv. 2025 Sep 8;9:100417. doi: 10.1016/j.ijnsa.2025.100417 (PMC12482300; doi:10.1016/j.ijnsa.2025.100417)
Supplement: Supplementary file 1 [file mmc1.docx]

**Supplementary 3 – Type of operation, LOS & Delayed discharge of included studies**

| **Study** | **Author/ publication year/ country** | **Type of operation/**  **Time of operation** | **Study type** | **Sample size** | **Delayed discharge rate**  **Number, (%)** | **LOS of**  **Delayed group**  **Median (day)** | **LOS of**  **Non-Delayed group**  **Median (day)** | **Note** |
| --- | --- | --- | --- | --- | --- | --- | --- | --- |
| 1. Prolong postoperative ileus (PPOI) in colorectal surgery within Enhanced Recovery Protocol: A multivariate analysis | Kocian P, Whitley A.  2022  Czech Republic | Colorectal resections;  -113 pts had right hemicolectomies  -107 pts had left Hemicolectomies  -152 pts had rectal resections  -2 pts had total colectomies  *(85 pts had mini-invasive procedure; 32 laparoscopic, 53 robotic procedure)  *289 pts had laparotomy  *77 pts had stoma | -Retrospective cohort study  -ERAS | 374 pts | 62 pts (16.6%) | 13 | 9 | PPOI, which is associated with delayed discharge |
| 2. Optimizing discharge decision making in colorectal surgery: a prospective cohort study of discharge practices in a recently implemented enhanced recovery pathway | Caminsky NG, Hamad D, He BH, Zhao K, Al Mahroos M, Feldman LS, et al.  2021  Canada | -51 % underwent Laparoscopic  - 49% Laparotomy  -13% had stoma formation | -Prospective cohort study  -ERAS | -73 pts | - 28 pts (37%) remained in hospital after discharged criteria were met ( DHD). | 6 (4–8) | 5 (3-8) |  |
| 3. The influence of peri-operative factors for accelerated discharge following laparoscopic colorectal surgery when combined with and enhanced recovery after surgery (ERAS) pathway | Chand M, De’Ath HD, Rasheed S, Mehta C, Bromilow J, Qureshi T.  2016  The United Kingdom (UK) | -The most common operation was laparoscopic anterior resection (n= 123, 41%).  -Rt hemicolectomy 101 (33%)  -Lt hemicolectomy 8 (2.7%)  -Ileocaecal resection 12 (4)  -panproctocolec  Tomy 8 (2.7)  -sigmoid colectomy 6(2%)  -subtotal colectomy 6 (2%)  -total colectomy 6(2%)  Hartmann’s 3(1%) | - Prospective cohort study  - ERAS | - 300 pts | - 115 pts (38%) discharged after 72 h (DHD) | 4.8 | 3 |  |
| 4. Patient outcomes related to in-hospital delays in Appendectomy for appendicitis: A retrospective study | Claydon O, Down B, Kumar S.  2022.  UK | -Appendectomy  -137 pts (30.7%), "time to surgery" was under 12 hours  - 309 pts (69.3%) "time to surgery" was over 12 hours. | -Retrospective cohort study | - 446 pts | - 319 pts (71.5%) |  |  |  |
| 5. Compliance with enhanced recovery protocols in elderly patients undergoing colorectal resection | Hallam S, Rickard F, Reeves N, Messenger D, Shabbir J.  2018.  UK | -Colorectal surgery | -Retrospective  -ERAS | 294 pts |  | > 6 days  (DHD defined as LOS >6 days) | 6 (5-8) |  |
| 6. Risk of delayed discharge and re-operation of gastric bypass patients with Psychiatric comorbidity – a nationwide cohort study | Lagerros YT, Brandt L, Sundbom M, Hedberg J, BodÃ©n R.  2020  Sweden | -Gastric Bypass | -Retrospective | - 22,539 pts | 9,480 pts (42.06%) |  |  |  |
| 7. Pre-operative factors prolong the length of stay in elective colorectal surgery | Ngui NK, Hitos K, Ctercteko G.  2011  Australia | -Elective colorectal resection | -Retrospective cohort study | 161 pts | 21 (13%) | 15 | 8 |  |
| 8. Predicting delayed discharge in a multimodal enhanced recovery pathway | Keller DS, Tantchou I, Flores-Gonzalez JR, Geisler DP.  2017  The United State of America (USA) | -Elective laparoscopic Colorectal surgery | -Retrospective  -ERAS | 274 pts | -45 pts (16.42%) | 6 and more | 5 and less |  |
| 9. Predicting who will fail early discharge after laparoscopic colorectal surgery with an established enhanced recovery (ERP)pathway | Keller DS, Bankwitz B, Woconish D, Champagne BJ, Reynolds HL, Stein SL, et al.  2014  USA | - Laparoscopic Colorectal surgery  -every hour of operating time increased the risk of length of stay > 4 days by 2.35 %. | -Retrospective  -ERP | 548 pts | 273 (48.9)% | 4 and longer | 3 |  |
| 10. Accelerated discharge within 72 hours of colorectal cancer resection using simple discharge criteria | Emmanuel A, Chohda E, Botfield C, Ellul J.  2017.  UK | Colorectal resection (90% has Laparoscopic procedures) | -Retrospective cohort study  -Simple discharge criteria | -256 pts | - 107 (42%) | 12 | 3.3 | Median LOS was 3 days |
| 11. Factors predicting deviation from an enhanced recovery program (ERP) and delayed discharge after laparoscopic colorectal surgery | Boulind CE, Yeo M, Burkill C, Wit A, James E, Ewings P, et al.  2012  UK | -Elective laparoscopic colorectal resection  -15 pts (9%) were converted from laparoscopic to laparotomy  -164pts(91%) remained laparoscopic | -Retrospective cohort study  -ERAS | -176 pts | 64 pts (36%)) |  |  | Median LOS was 5 days |
| 12. Patient’s refusal as major limitation of early discharge after colorectal resection in an enhanced recovery program | Collard MK, Anyla M, Lefevre JH, Shields C, Laforest A, Gutton C, et al.  2020.  France | 283 pts had Colorectal resection  -231 (82%) had colectomy  -51(18%) had proctectomy | -Prospective cohort study  -ERAS | -283 pts | -52% had DHD  (discharged after 5 days) | > 5 | 3-5  (Mean LOS was 5 days (2–33) days). | - A total of 136 patients (48%) were discharged at 3-/5-day, within 9 pts were readmitted (3%). |
| 13. Deviation from a clinical pathway post pancreatoduodenectomy predict 90 day unplanned re-admission | Karunakaran M, Barreto SG, Singh MK, Kapoor D, Chaudhary A.  2020.  India | Pancreatoduodenectomy | -Retrospective analysis  -Clinical Pathway | 162 pts | -deviations from the pathway were frequent (91%) |  |  | Mean LOS 10.8 +/_5.8 days |
| 14. Early Red flags associated with delayed discharge in patients undergoing Gastrectomy: Analysis of perioperative variables and ERAS protocol items | Parise P, Cinelli L, Ferrari C, Cossu A, Puccetti F, Garutti L, et al.  2020  Italy | Gastrectomy  -Total gastrectomy (62.8%)  -Distal gastrectomy (37.2%) | -Retrospective cohort study  -ERAS | -180 pts |  |  |  | Targeted LOS was set to 9 post operative days |
| 15. Prospective evaluation of discharge trends after colorectal surgery with ERAS pathway | Slieker JC, Clerc D, Hahnloser D, Demartines N, HÃ¼bner M.  2017.  Switzerland | Elective Colorectal surgery, or surgery > 24 hours after an unplanned admission. Immediate emergency (0-24 h were excluded).  -63 colectomies  -16 rectal resection  -35 stoma creation or closure | -Prospective study  -ERAS | 114 consecutive pts | - -70% had DHD  - Only 30% of patients went home on the day that all discharge criteria were met. | -day of d/c  Median (range)  5 (3-7) Overall, patients were discharged at a median of 2 days (interquartile range 1–3) after fulfillment of discharge criteria. |  | DHD = the time laps between the time that all criteria met and the time of actual hospital d/c |
| 16. Deviation and failure of enhanced recovery after surgery following laparoscopic colorectal surgery: early prediction model | Smart NJ, White P, Allison AS, Ockrim JB,  2012  UK | Elective Laparoscopic colorectal surgery | -Retrospective cohort study  -ERAS | -385 pts | -122 (31%) patients stayed longer than 1 week (delayed discharge) and 159 (41%) deviated in up to two postoperative ERAS factors | > 8 days; | - median LOS of 6 days; | DHD = prolonged LOS >8 days  LOS=Time spent in hospital postoperatively  ERAS failue=DHD |
| 17. Enhanced recovery after surgery in colon and rectal surgery: identification of predictive variables of failure in a monocentric series including 733 patients | Vignali A, Elmore U, Guarneri G, De Ruvo V, Parise P, Rosati R.  2021  Italy | -Laparoscopic 92%,  -converted to open surgery 8%  -75% of patients had Colon resection  -22.9% had rectum resection  -2.1% ad proctocolectomy | -Retrospective cohort study  -ERAS | -733 pts | 320 pts (**43.7%**) had an LOS greater than ideal threshold for discharge  -413 pt (56.3%) had LOS lower or equal to the ideal threshold for d/c of the specific group | -median LOS was 5 days (2-145 days) for colon  - median LOS was7 days (3-40 days) for rectal resection  - median LOS was11 (5-21 days) for proctocolectomy |  | -Idea POD =5 days for colonic, POD 7 for anterior resection, POD 11 for proctocolectomy |
| 18. Focused preoperative patient stoma education prior to ileostomy formation after anterior resection, contributes to a reduction in delayed discharge within enhanced recover program | Younis J, Salerno G, Fanto D, Hadjipavlou M, Chellar D, Trickett JP.  2012.  UK | Anterior resection with the formation of a loop ileostomy | -Quasi-experimental study  -Compared the non ERP group and ERP group with comprehensive stoma education | -240 pts  (120 before ERP,120 after ERP with stoma education) | -21 patients in the pre-ERP group (17.5%) experienced postponed hospital discharge,  0.8% in post ERP group had DHD | -Average length of hospital stay was 14 days (7-25 days) prior ERP introduction. |  | -The mean length of stay amongst the ERP patients was 8 days, ranging from 3 to 17 days. |
| 19. Why still in hospital after laparoscopic colorectal surgery within an enhanced recovery program? | Munk-Madsen P, Eriksen J, Kehlet H, Gogenur I.  2019  Denmark | -Elective laparoscopic colorectal cancer resection  -4pt conversed to open surgery | -Analytic cross-section study  -ERAS | -96 pts |  | - The median LOS for the whole group was 3 days (range 1–14). |  | -LOS of pt. with stoma was 5day, no stoma was 2 days  -LOS of open surgery=6 days (4-7) |
| 20. Pre -and postoperative stoma education and guidance within an enhanced recovery after surgery program reduce length of hospital stay in colorectal surgery | Forsmo HM, Pfeffer F, Rasdal A, Sintonen H, Korner H, Erichsen C.  2016  Norway | Colorectal surgery | -Randomised controlled trial  -ERAS | 122 pts  -61 extended stoma program  -61 current stoma education program |  | Total LOS was significantly shorter in the ERAS group with extended stoma education than the standard stoma education care group (median range 6 days vs. 9 days |  |  |
