# Supplementary material for: Improving delayed discharge in gastrointestinal surgery patients: An integrative review
Source: Int J Nurs Stud Adv. 2025 Sep 8;9:100417. doi: 10.1016/j.ijnsa.2025.100417 (PMC12482300; doi:10.1016/j.ijnsa.2025.100417)
Supplement: Supplementary file 2 [file mmc2.docx]

**Supplementary 2: Studies included in the review.**

| **Study, authors, publication year, and country** | **Aim (A), intervention (I), Method (M)** | **Setting (S) and sample size (SS)** | **Summary of the results** | **Limitation (L) and Suggested future research (S)** |
| --- | --- | --- | --- | --- |
| 1. Prolong postoperative ileus in colorectal surgery within Enhanced Recovery Protocol: A multivariate analysis  Kocian P, Whitley A.  2022.  Czech Republic | A: (1) to identify factors influencing prolonged postoperative ileus (PPOI) of colorectal surgery under ERAS protocol (2) to identify the relation of PPOI and hospital length of stay (LOS).  I: none  M: reviewed a prospectively managed database of the patents | S: The department of surgery, Motal University hospital, Prague  SS: 374 patients who underwent colorectal resections between April 2016 and May 2020. | - 62 patients (16.6%) developed PPOI  - factors influence major abdominal surgery, late feeding, late mobility post-surgery  - PPOI was associated with a longer average LOS and a higher rate of hospital site infection | L: the study was limited by retrospective design and relatively small number of patients |
| 2. Optimizing discharge decision making in colorectal surgery: a prospective cohort study of discharge practices in a recently implemented enhanced recovery pathway  Caminsky NG, Hamad D, He BH, Zhao K, Al Mahroos M, Feldman LS, et al.  2021  Canada | A: (1) to compare time to readiness for discharge by set criteria and actual length of stay (LOS) in a newly implemented colorectal enhanced recovery pathway and (2) to identify reasons for delayed hospital discharge  I: none  M: (1) review patient's medical file (2) interview treating team | S: A university teaching hospital in Montreal, Canada  SS: 73 adult patients (age 67 ± 14 years, 56% men, 51% laparoscopic, 13% stoma creation) undergoing elective colorectal surgery in a university hospital with a recently implemented recovery pathway | - Median LOS was 6 (4–8) days and median time to readiness for discharge was 5 (3–8) days (P < 0.001).  - Twenty-eight patients (37%) remained in hospital after DC was achieved.  - Reasons delayed discharges were medically justified (e.g., workup [13%] or treatment of complications not captured by DC [2.6%]), unnecessary hospital stays were common (e.g., perceived need for observation [16%], or patients not willing to be discharged [11%] | L: Data were collected in a single institution with a newly implemented ERP and, for this reason, the results may not be generalizable to institutions with well-established pathways or traditional care settings. Also, the study used a prospective systematic approach to record reasons for delayed hospital discharge; however, these reasons sometimes overlapped and could not be clearly defined. |
| 3. The influence of peri-operative factors for accelerated discharge following laparoscopic colorectal surgery when combined with and enhanced recovery after surgery (ERAS) pathway  Chand M, De’Ath HD, Rasheed S, Mehta C, Bromilow J, Qureshi T. 2016  The United Kingdom  (UK) | A: to evaluate the early outcomes of patients undergoing such a regime to determine which peri-operative factors may predict safe accelerated discharge.  I: none  M: Data were prospectively collected on consecutive patients undergoing laparoscopic colorectal surgery. | S: a single institution in the United Kingdom  SS: 300 patients were included in the analysis. All patients were followed up from admission until discharge and monitored for re-admission. Surgery was performed by a single experienced laparoscopic surgeon (TQ). | The most common operation was laparoscopic anterior resection (n ¼ 123, 41%).  - Mean length of stay was 4.8 days (standard deviation 5.9), with 185 (62%) patients discharged within 72 h.  - Ten (3%) patients had a post-operative complication.  - Three independent predictors of delayed discharge were identified; BMI (OR 1.06, 95%CI 1.01e1.11), operation length (OR 0.99, 95%CI 0.98e0.99) and complications (OR 16.26, 95%CI 4.88e54.08). | L: The factors which reached statistical significance should be considered with caution. Specifically, the OR of 1.06 and 0.99 (BMI and length of operation) are of little clinical significance. Although the complication rate would be the most intuitive factor, the confidence interval was large. This might suggest an underpowered study. The study findings are also limited to a single centre, and a single surgeon, and hence may not be widely applicable to other centres. |
| 4. Patient outcomes related to in-hospital delays in Appendectomy for appendicitis: A retrospective study  Claydon O, Down B, Kumar S.  2022  UK | A: to determine if a delay in laparoscopic appendicectomy in cases of acute appendicitis of over 12 hours is related to delayed discharge from the hospital.  I: none  M: review of the medical records of patients who underwent laparoscopic appendicectomy for appendicitis between 01/01/2018 and 30/08/2019. | S: 550 beds of a UK district general hospital  SS: 446 patients were included in the study. | - In 137 patients (30.7%), "time to surgery" was under 12 hours; in 309 patients (69.3%) "time to surgery" was over 12 hours.  - 319 patients (71.5%) had a delayed discharge; 303 patients (67.9%) had complicated appendicitis, and 143 patients had severe appendicitis (32.1%). No statistically significant association between "time to surgery" and delayed discharge, appendicitis severity, conversion, or 30-day re-presentations was observed. | L: the relatively small sample size and the fact that a singlecentre study may not be representative of the characteristics of the broader population or common management procedures in other institutions. More importantly, we acknowledge that our study did not take into account the preoperative clinical rationale regarding the timing of appendicectomy in each case. |
| 5. Compliance with enhanced recovery protocols in elderly patients undergoing colorectal resection  Hallam S, Rickard F, Reeves N, Messenger D, Shabbir J.  2018  UK | A : to evaluate the feasibility of ERAS in patients of all ages undergoing colorectal surgery  I: none  M: A prospective database of a consecutive series of patients undergoing colorectal resections with ERAS between August 2012 and December 2014 was evaluated. Patients were divided into four age groups. Outcomes studied were compliance with ERAS elements, LOS, morbidity and mortality | S: University Hospitals Bristol NHS Foundation Trust,  SS: 294 patients in the study cohort. 79 were < 60 years, 81 were 60–69 years, 86 were 70–79 years and 48 were ≥80 years of age. | - There was no significant difference between age groups in compliance with ERAS elements.  - Age was not predictive of delayed discharge (LOS >6 days) or morbidity.  - Factors that were predictive of delayed discharge on multivariate analysis were open surgery (odds ratio [OR]: 2.23, p=0.003), conversion to open surgery (OR: 3.23, p=0.017), stoma formation (OR: 2.10, p=0.019) and chronic obstructive pulmonary disease (OR: 4.12, p=0.038).  - Factors predictive of morbidity on multivariate analysis comprised conversion to open surgery (OR: 7.72, p=0.004), high creatinine (OR: 1.03 per unit increase in creatinine, p=0.008) and stoma education (OR: 0.31, p=0.030). | Not mentioned |
| 6. Risk of delayed discharge and re-operation of gastric bypass patients with Psychiatric comorbidity – a nationwide cohort study  Lagerros YT, Brandt L, Sundbom M, Hedberg J, BodÃ©n R.  2020.  Sweden | A: to investigate whether a psychiatric diagnosis before GBP surgery is associated with delayed discharge and rate of reoperation in a nationwide Swedish cohort.  I: none  M: Patients undergoing GBP surgery during 2008–2012 were identified and followed up through the National Patient Register and the Prescribed Drug Register. | S: nationwide Sweden  SS: 22,539 patients identified, a prior diagnosis of bipolar disorder, schizophrenia, depression, neurotic disorders, ADHD , substance use disorder, eating disorder, personality disorder, or self-harm | 9480 patients were found to be associated with delayed discharge after GBP surgery (odds ratio [OR] = 1.47, confidence interval [CI] 1.34–1.62), especially in patients with psychiatric hospitalization exceeding 1 week in the 2 years preceding GBP surgery (OR = 2.06, CI 1.30–3.28), compared with those not hospitalized within psychiatry. | L: the study was only able to identify those with diagnosed psychiatric illness, and we were restricted to the diagnoses made by physicians in hospital inpatient and outpatient care, as diagnoses made by primary care physicians are not included in the National Patient Register. |
| 7. Pre-operative factors prolong the length of stay in elective colorectal surgery  Ngui NK, Hitos K, Ctercteko G.  2011.  Australia | A: to identify which preoperative factors are associated with non-medical reasons for a delay in discharge and prolonged length of stay (LOS) in the hospital after elective colorectal resections  I: nil  M: a retrospective review of prospectively collected data was performed on all the elective colorectal resections | S: Westmead Hospital  SS: 161 patients underwent an elective colorectal resection between 1 January 2007 to 31 December 2008 and met the inclusion criteria. | - Overall median age was 66 years (58–75 years).  - Median post-operative LOS for patients not delayed in discharge was 8 days and 15 days for patients with an identifiable non-medical reason for delay (P < 0.0001).  - Preoperative factors significantly associated with a delay discharge included **advanced age** (odds ratio (OR): 10.5; 95% confidence interval (CI): 3.0–37.7; P < 0.0001), being **widowed** (OR: 3.5; 95% CI: 1.2–10.2; P = 0.02) and **living in a retirement village** (OR 15.4; 95% CI: 1.6–150.3; P = 0.019). Higher ASA scores strongly correlated with longer LOS. | L: the retrospective nature of the study, these findings will form the basis of a prospective cohort study that will examine the effect of a discharge planner consultation on patients at the preadmission clinic who are judged to be high risk of having a delay in discharge because of non-medical reasons, from the preoperative factors identified in these results. |
| 8. Predicting delayed discharge in a multimodal enhanced recovery pathway    Keller DS, Tantchou I, Flores-Gonzalez JR, Geisler DP.  2017.  The United States of America (USA) | A: to identify the patient and procedural variables associated with delayed discharge despite an established ERP.  I: none  M: a prospectively-maintained departmental database was retrospectively reviewed | S: No setting details informed on the article.  SS: Between 8/1/13 and 7/31/15, 301 patients were evaluated. 274 patients met the inclusion criteria, | - 229 successes and 45 failures. - Groups were similar in demographics. Failures had higher rates of preoperative anxiety(p ¼ 0.0352), chronic pain(p ¼ 0.0040), prior abdominal surgery(p ¼ 0.0313), and chemoradiation(p ¼ 0.0301).  - Intraoperatively, failures had higher conversion rates (13.3% vs. 1.7%, p ¼ 0.0002), transfusions(p ¼ 0.0032), and longer operative times(219.8 vs. 183.5min,p ¼ 0.0099).  - Total costs for failures were higher than successes ($22,127 vs. $13,030,p ¼ 0.0182).  - Variables independently associated with failure were anxiety (OR 2.28, p ¼ 0.0389), chronic pain(OR 10.03, p ¼ 0.0045), and intraoperative conversion(OR 8.02, p ¼ 0.0043). | S: While not modifiable before surgery, practice patterns can also be changed so patients that undergo intraoperative conversion are met with aggressive reconditioning and postoperative discharge planning. These could have an immediate physical therapy consultation on postoperative day 1, with resulting social work consultation for rehabilitation or home care as needed, to expedite the appropriate discharge process. Future study will evaluate the implementation process and impact of these measures |
| 9. Predicting who will fail early discharge after laparoscopic colorectal surgery with an established enhanced recovery (ERP)pathway  Keller DS, Bankwitz B, Woconish D, Champagne BJ, Reynolds HL, Stein SL, et al.  2014  USA | A: to identify predictors for patients who might fail early discharge despite being under ERP protocol  I: none  M: A prospectively maintained database was reviewed for major elective laparoscopic colorectal surgical procedures. Cases were divided into day of discharge groups: B3 days and [4 days. All followed a standardized ERP. | S: Division of Colorectal Surgery, Department of Surgery, University Hospitals-Case Medical Center  SS: 548 cases included in the analysis. There were 275 patients (50.2 %) in the B3-days group and 273 (49.8 %) patients in the [4-days group. The main indications for surgery in the B3-days and[4-days patients were colorectal cancer (41 and 53 %) and diverticulitis (21 and 13 %), respectively | There were 275 less than or equal to 3 days patients and 273 greater than 4 days patients. There were significant differences between groups in body mass index (p = 0.0123), comorbidities (p = 0.0062), ASA class (p = 0.0014), operation time (p\0.001), postoperative complications (p\0.001), and 30-day reoperation rate (p = 0.0004). There were no significant differences for intraoperative complications (p = 0.724), readmissions (p = 0.187), or mortality rate (p = 1.00). Significantly more patients were discharged directly home in the B3-days cohort. Using logistic regression, every hour of operating time increased the risk of length of stay [4 days by 2.35 %. | L: the study population was not randomised, so our results could be affected by group selection bias. The study also was a retrospective review, so we were limited to a database and chart review. We would not be aware of any factors that affect the length of stay or discharge planning that are not written in the medical record.  S: A prospective, randomised study with matched patient groups would address these limitations |
| 10. Accelerated discharge within 72 hours of colorectal cancer resection using simple discharge criteria  Emmanuel A, Chohda E, Botfield C, Ellul J.  2017.  UK | A: to evaluate the safety and feasibility of accelerated discharge within 72 hours of all elective colorectal cancer resections using simple discharge criteria.  I: none  M: Elective colorectal cancer resections performed between August 2009 and December 2015 by a single surgeon were reviewed. Perioperative care was based on an enhanced recovery programme. A set of simplified discharge criteria were used. Outcomes including postoperative complications, readmissions and reoperations were compared between patients discharged within 72 hours and those with a longer postoperative stay. | S: King's College Hospital  SS: 256 colorectal cancer resections (90% laparoscopic) were performed. The mean patient age was 70.8 years. | The mean patient age was 70.8 years. The median length of stay was 3 days. Fifty-eight per cent of all patients and sixty-three per cent of patients undergoing laparoscopic surgery were discharged within 72 hours  Accelerated discharge was not associated with adverse outcomes compared with delayed discharge. Patients discharged within 72 hours had significantly fewer postoperative complications, readmissions and reoperations. Open surgery and stoma formation were associated with discharge after 72 hours but not age, co-morbidities, neoadjuvant chemoradiation or surgical procedure. | L: retrospective design and the associated risk of selection bias. Moreover, it involves a single surgeon’s experience, which may not be generalisable.  S: Future research should focus on the potential cost benefit to achieving early discharge for large proportions of patients. More patients could benefit from early discharge with improvements in processes such as access to outpatient advice and review for minor complications, and preoperative education and training for stoma care. |
| 11. Factors predicting deviation from an enhanced recovery program and delayed discharge after laparoscopic colorectal surgery  Boulind CE, Yeo M, Burkill C, Witt A, James E, Ewings P, et al.  2012.  UK | A: to identify factors that predict postoperative deviation from an enhanced recovery program (ERP) and/or delayed discharge following colorectal surgery.  I: none  M: Data were prospectively collected from all patients undergoing elective laparoscopic colorectal resection between January 2006 and December 2009. | S: Yeovil District Hospital  SS: 176 patients (90 women) of mean age 68 years who underwent elective laparoscopic colorectal resection (including right, left or subtotal colectomy, segmental resection, sigmoid colectomy and rectal resection) for benign and malignant disease. | - The most common reason was failure to mobilize, which often occurred in conjunction with paralytic ileus or analgesic failure.  - Factors independently predicting ERP deviation on multivariate analysis were pathology and intra-operative complications.  - The median length of stay was 5 days. Sixty-four (36%) patients had a prolonged length of stay that was predicted by age, number of procedures and ERP deviation | nil |
| 12. Patient’s refusal as major limitation of early discharge after colorectal resection in an enhanced recovery program  Collard MK, Anyla M, Lefevre JH, Shields C, Laforest A, Gutton C, et al. 2020.  France | A: to evaluate the feasibility and safety of a 3-day hospitalization after colectomy and 5-day hospitalization after proctectomy in the setting of an ERAS program.  I: ERAS program  M: An ERAS program was prospectively proposed to all patients who required a colorectal resection (January 2014– December 2018) with a 3- or 5-day discharge objective. The success of the program was defined by a 3-/5-day hospitalization without complications and without readmissions. | S : No mention of setting name.  SS: Among 283 patients included, 232 patients had a colectomy (82%) and 51 (18%) patients a proctectomy | Eighty-six patients experienced complications (30%) including fifteen severe complications (5%). Mean hospital stay was 5.1 ± 3.7 (2–33) days. A total of 136 patients (48%) were discharged at 3-/5-day, within 9 were readmitted (3%). Discharge was delayed after 3-/5- day for complications (n = 65, 23%), CRP > 120 (n = 45, 16%) or refusal without medical reason (n = 37, 13%).  The success rate of the program was 45% (n = 127). | nil |
| 13. Deviation from a clinical pathway post pancreatoduodenectomy predict 90 day unplanned re-admission  Karunakaran M, Barreto SG, Singh MK, Kapoor D, Chaudhary A.  2020.  India | A: To determine the frequency and relevance of deviations from a post pancreatoduode-  nectomy (PD) clinical care pathway.  I: nil  M: A retrospective analysis using a prospectively maintained database of a post-PD clinical care pathway was carried out between May 2016 and March 2018. | S: The Department of Gastrointestinal Surgery, Medanta, The Medicity, Gurgaon, a tertiary referral care center,  SS: total 162 pts Group 1 -No deviation, n = 14 Group 2 - Deviation in 1- 4 factors, n = 84 Group 3 - Deviations in 5 -8 factors, n =64 | Post-PD clinical care pathways are feasible but deviations from the pathway are frequent (91%). An increase in frequency of deviations from the pathway was significantly associated with increased risk of POPF and delayed gastric emptying, delayed discharge, risk of mortality and 90-day unplanned re-admission rate | S: The next step would be to revisit the clinical pathway to revise it to improve the outcomes of our patients, especially targeting the patients in Group 3. |
| 14. Early Red flags associated with delayed discharge in patients undergoing Gastrectomy: Analysis of perioperative variables and ERAS protocol items  Parise P, Cinelli L, Ferrari C, Cossu A, Puccetti F, Garutti L, et al.  2020.  Italy | A: to assess whether perioperative variables or deviation from ERAS items is associated with delayed discharge after gastrectomy  I: none  M: All clinical data were retrospectively collected from a prospectively maintained database | . S: Department of Gastrointestinal surgery, San Raffaele Hospital, Milan.  SS: 108 patients who underwent Gastrectomy surgery under ERAS Protocol between August 2014 and December 2017. | Multivariate regression analysis revealed that incomplete immunonutrition, failure to extubate the patient at the end of surgery, intraoperative crystalloids [2150 ml and blood transfusion [268 ml, surgery duration[195 min, and failure to mobilise patients within 24 h from surgery were associated with delayed discharge. | L: The small sample size and the lack of a control group outside the ERAS framework may prevent the correct identification of variables associated with delayed discharge.  S: future studies should have a bigger sample size and involve multiple setting |
| 15. Prospective evaluation of discharge trends after colorectal surgery with ERAS pathway  Slieker JC, Clerc D, Hahnloser D, Demartines N, HÃ¼bner M.  2017.  Switzerland | A: to assess the timing of complete medical recovery in relation to the timing of actual discharge, and to assess reasons for prolonged hospital stay within an ERAS pathway  I: none  M: Fulfillment of discharge criteria was assessed daily and reasons for prolonged hospital stay were documented. | S: Department of Visceral Surgery, University Hospital CHUV, Lausanne  SS: 104 consecutive patients undergoing elective colorectal surgery within an ERAS pathway were included in this prospective analysis. | - Only 30% of patients went home on the day that all discharge criteria were met. Overall, patients were discharged at a median of 2 days (interquartile range 1–3) after fulfillment of discharge criteria.  - Reasons for delayed discharge were (1) organizational in 20%; (2) patient or surgeon unwilling in 29%; and (3) because the patient was deemed to be discharged too soon distance from the operation in 51%. | L: A limitation of this study was that discharge criteria were documented for every patient daily until POD 9 in colon surgery and POD 10 in rectum surgery but not until hospital discharge for patients hospitalized longer. |
| 16. Deviation and failure of enhanced recovery after surgery following laparoscopic colorectal surgery : early prediction model  Smart NJ, White P, Allison AS, Ockrim JB,  2012  UK | A: to investigate factors associated with delayed discharge and to produce a predictive scoring system for ERAS failure.  I: none  M: A retrospective review was carried out of case notes of patients who underwent elective laparoscopic colorectal resection and ERAS between 2002 and 2009 | S: Yeovil District Hospital  SS: 385 patient records were reviewed | - median length of stay of 6 days; 122 (31%) patients stayed longer than 1 week (delayed discharge) and 159 (41%) deviated in up to two postoperative ERAS factors.  - Patient demographic factors were not predictive of delayed discharge.  - Deviation from ERAS factors at the end of the first postoperative day, including continued intravenous fluid infusion, lack of functioning epidural, inability to mobilize, vomiting requiring nasogastric tube insertion and re-insertion of urinary catheter, were strongly associated with delayed discharge.  - A five-element predictive scoring system for ERAS failure and delayed discharge was formulated | nil |
| 17. Enhanced recovery after surgery in colon and rectal surgery: identification of predictive variables of failure in a monocentric series including 733 patients  Vignali A, Elmore U, Guarneri G, De Ruvo V, Parise P, Rosati R.  2021.  Italy | A: To identify factors associated with early deviation and delayed discharge within an Enhanced Recovery after Surgery (ERAS) pathway.  I: none  M: a retrospective review of prospectively collected data of consecutive patients who underwent laparoscopic or open colorectal surgery and managed with a standardized ERAS pathway between April 2015 and October 2018. ERAS items were assessed within 48 h after surgery | S: San Raffaele Hospital  SS: 733 patients met the inclusion criteria. | Multivariate analysis showed that age≥75 years (P=0.02), ASA score≥3 (P=0.03), open surgery or conversion to open (P=0.001), non-compliance with the intra-operative balanced fluid therapy (P=0.049), failure to early removal of the urinary catheter (P=0.001), to discontinue IV fluid (P=0.02) and to early mobilization (P=0.001) were independently associated with ERAS failure. The generated score had a specificity of 84% and a positive predictive value of 72%. Patients who would have a length of stay longer than the median for each surgical procedure were properly identified (Area under ROC Curve=0.753, P | L: - small validation pool used to prospectively validate the score.  - retrospective nature of the analysis.  - another possible pitfall is the assumption that LOS reflects the outcome. At the current time, the only consensus that can reasonably be agreed upon is that discharge from hospital is not synonymous with recovery and may be unduly influenced by institutional factors that are independent of the effect of an ERAS program. |
| 18. Focused preoperative patient stoma education prior to ileostomy formation after anterior resection, contributes to a reduction in delayed discharge within Enhanced Recovery program (ERP)  Younis J, Salerno G, Fanto D, Hadjipavlou M, Chellar D, Trickett JP.  2012.  UK | A: to promote independent stoma management post-operatively, thus expediting hospital discharge.  I: Stoma comprehensive training prior to operation  M: Data collection on patients undergoing anterior resection with the formation of a loop ileostomy was carried out retrospectively prior to ERP (January 2006 to August 2008) and prospectively following the introduction of ERP (September 2008 to October 2010). | S: A single district general hospital  SS: Two hundred forty patients underwent elective anterior resection with the formation of a loop ileostomy, 120 prior ERP and 120 post-ERP. | Average length of hospital stay was 14 days before ERP introduction, with a range of 7–25 days. The mean length of stay amongst the ERP patients was 8 days (p=0.17), ranging from 3 to 17 days. Twenty-one patients in the pre-ERP group (17.5%) experienced postponed hospital discharge due to a delay in independent stoma management, compared to one patient experiencing such a delay after the introduction of ERP (0.8%, p<0.0001) |  |
|  |  |  |  |  |
| 19. Why still in hospital after laparoscopic colorectal surgery within an enhanced recovery program?  Munk-Madsen P, Eriksen J, Kehlet H, Gogenur I.  2019.  Denmark | A: to identify and describe factors compromising early postoperative recovery by asking ‘why is the patient still in hospital today?’ after laparoscopic colorectal cancer surgery within an enhanced recovery after surgery program  I: none  M: Patients undergoing elective laparoscopic colorectal cancer resection were evaluated postoperatively with predefined potential reasons for still being in hospital. The primary outcome was ‘reason for still being in hospital’ on postoperative day 0–4 and secondarily length of stay with a focus on differences between patients with and without a stoma | S: Zealand University Hospital Roskilde, Denmark,  SS: 96 patients having colorectal cancer surgery were included. | - The median length of stay for the whole group was 3 days (range 1–14).  - The four dominant causes for patients without a stoma to be in hospital were lack of gastrointestinal function, lack of early mobilization, lack of normal micturition and nausea. Patients with a stoma stayed in hospital due to stoma training, lack of gastrointestinal function, lack of free micturition and a miscellaneous ‘others’ group. | S: Together with a focus on frailty, future studies should focus on improving early mobilization, prevention and treatment of postoperative urinary retention and improved stoma care training to minimise delay in postoperative recovery and discharge. |
| 20. Pre -and postoperative stoma education and guidance within an enhanced recovery after surgery program reduce length of hospital stay in colorectal surgery  Forsmo HM, Pfeffer F, Rasdal A, Sintonen H, Korner H, Erichsen C. 2016.  Norway | A: To explore whether implementing an enhanced recovery after surgery (ERAS) program with specialized nurses focusing on stoma counselling and education can lead to shorter hospital stays, fewer readmissions, fewer stoma-related complications, and improved health-related quality of life (HRQoL) compared to the current standard stoma education in traditional care pathways.  I: Nurse-focused stoma counselling and education program | S: Haukeland University Hospital in Bergen, Norway  SS: 122 adult patients eligible for laparoscopic or open colorectal resection who received a planned stoma were treated in either the ERAS program with extended stoma education (n ¼ 61) or standard care with current stoma education (n ¼ 61). | Total hospital stay was significantly shorter in the ERAS group with education than the standard care group (median [range], 6 days [2 - 21 days] vs. 9 days [5- 45 days]; p < 0.001).  Regarding overall major and minor morbidity, re-admission rate, stoma-related complications and 30-day mortality, the two treatment groups exhibited similar outcomes. | L: the study did not measure days to stoma independence and proficiency. The reason for this large difference between the date of stoma proficiency and the date of discharge was not given. |
