# Supplementary material for: Improving delayed discharge in gastrointestinal surgery patients: An integrative review
Source: Int J Nurs Stud Adv. 2025 Sep 8;9:100417. doi: 10.1016/j.ijnsa.2025.100417 (PMC12482300; doi:10.1016/j.ijnsa.2025.100417)
Supplement: Supplementary file 3 [file mmc3.docx]

**Supplementary 1: Criteria for quality evaluation by JBI Sumari**

| **Criteria used for cohort study** | **Criteria used for randomised controlled trial study** |
| --- | --- |
| 1. Were the groups similar and recruited from the same population?  2. Were the exposures measured similarly to assign people to both exposed and unexposed groups?  3. Was the exposure measured in a valid and reliable way?  4. Were confounding factors identified?  5. Were strategies to deal with compounding factors stated?  6. Were the groups/participants free of the outcome at the start of the study (or at the moment of exposure)?  7. Were the outcomes measured in a valid and reliable way?  8. Was the follow-up time reported and sufficient to be long enough for outcomes to occur?  9. Was follow-up completed, and if not, were the reasons to loss to follow-up described and explored?  10. Were strategies to address incomplete follow-up utilised?  11. Was appropriate statistical analysis used? | 1. Was true randomisation used for assignment of participants to treatment groups?  2. Was allocation to treatment groups concealed?  3. Were treatment groups similar at the baseline?  4. Were participants blind to treatment assignment?  5. Were those delivering treatment blind to treatment assignment?  6. Were outcomes assessors blind to treatment assignment?  7. Were treatments groups treated identically other than the intervention of interest?  8. Was follow up complete and if not, were differences between groups in terms of their follow up adequately described and analysed?  9. Were participants analysed in the groups to which they were randomised? |
| **Criteria used for analytical-cross section study** | **Criteria used for randomised controlled trial study (continued)** |
| 1. Were the criteria for inclusion in the sample clearly defined?  2. Were the study subjects and the setting described in detail?  3. Was the exposure measured in a valid and reliable way?  4. Were objective, standard criteria used for measurement of the condition?  5. Were confounding factors identified?  6. Were strategies to deal with confounding factors stated?  7. Were the outcomes measured in a valid and reliable way?  8. Was appropriate statistical analysis used? | 10. Were outcomes measured in the same way for treatment groups?  11. Were outcomes measured in a reliable way?  12. Was appropriate statistical analysis used?  13. Was the trial design appropriate, and any deviations from the standard RCT design (individual randomisation, parallel groups) accounted for in the conduct and analysis of the trial? |
| **Criteria used for quasi-experimental study** | |
| 1. Is it clear in the study what is the 'cause' and what is the 'effect' (i.e., there is no confusion about which variable comes first)?  2. Were the participants included in any comparisons similar?  3. Were the participants included in any comparisons receiving similar treatment/care, other than the exposure or intervention of interest?  4. Was there a control group?  5. Were there multiple measurements of the outcome both pre and post the intervention/exposure?  6. Was follow up complete and if not, were differences between groups in terms of their follow up adequately described and analysed?  7. Were the outcomes of participants included in any comparisons measured in the same way?  8. Were outcomes measured in a reliable way?  9. Was appropriate statistical analysis used? | |
